# Error in Figure 2B

**DOI:** 10.1001/jamanetworkopen.2022.16068

**Published:** 2022-05-18

In the Original Investigation titled “Evaluation of Budesonide-Formoterol for Maintenance and Reliever Therapy Among Patients With Poorly Controlled Asthma: A Systematic Review and Meta-analysis,”^1^ published March 1, 2022, the label of Figure 2B was changed from “SMART vs same GINA step 3” to “SMART vs same GINA step 3 or 4.” This article has been corrected.^1^
